# Interoceptive awareness in attention deficit hyperactivity disorder

**DOI:** 10.1371/journal.pone.0205221

**Published:** 2018-10-12

**Authors:** Jan R. Wiersema, Elke Godefroid

**Affiliations:** Department of Experimental Clinical and Health psychology, Faculty of Psychology and educational sciences, Ghent University, Ghent, Belgium; Anglia Ruskin University, UNITED KINGDOM

## Abstract

ADHD is considered a disorder of self-regulation. Recent research has shown that awareness of bodily states, referred to as interoceptive awareness, crucially contributes to self-regulatory processes. Impaired self-regulation in ADHD has been explained in terms of arousal regulation deficits in ADHD (the state regulation deficit (SRD) account). There is now ample support for the SRD account, however the exact reason for arousal regulation difficulties is not yet known. The SRD account explicitly refers to the ability to monitor one’s momentary bodily state as a prerequisite for effective state regulation. However, surprisingly, no study to date has tested the ability to become aware of bodily signals, i.e. interoceptive awareness, in ADHD. In the current study, we therefore compared interoceptive awareness between 24 adults with ADHD and 23 controls by means of both an objective (heartbeat perception task) and subjective measure (questionnaire) of interoceptive awareness. Results revealed a strikingly similar performance for both groups on both measures, suggesting preserved interoceptive awareness in adult ADHD.

## Introduction

Attention-deficit/hyperactivity disorder (ADHD) [1] is a common neurodevelopmental disorder characterized by symptoms of inattention, and/or hyperactivity and impulsivity, which often persists into adulthood [2,3]. ADHD leads to impairments in social and cognitive functioning in an array of settings. Several etiological models have been introduced in the literature and although they all have their own focus, they have in common that they consider ADHD as a disorder of self-regulation, comprising dysregulation of behavior, cognition, and emotions [4–8].

Self-regulation is the ongoing, dynamic, and adaptive modulation of internal state (emotion, cognition) or behavior, by oneself, mediated by central and peripheral physiology [9], and involves a complex interplay of psychological and physiological processes. The idea that bodily processes are crucially involved in self-regulation is not new but recently there is a renewed interest among researchers in the contribution of interoceptive awareness (IA) to emotion, cognition and behavior and how it might be linked to psychopathology. Interoception is the perception of autonomic bodily signals, with IA referring to the awareness of these signals [10]. The anterior insula, as the proposed locus of bodily awareness [11], is heavily involved in interoceptive processes [12–15]. IA has been shown to play a key role in the processing and regulation of emotions, with higher IA being associated with more intense feelings during emotion processing and higher activation of underlying brain regions, and better emotion regulation skills [16,17]. IA also contributes to other cognitive functions required for effective self-regulation such as selective attention, decision-making [18], memory [19], and error processing [20–22]. All these cognitive functions as well as emotion regulation have been reported to be disturbed in ADHD [23–28]. Moreover, ADHD has been associated with structural and functional insula abnormalities [29,30]. It is therefore surprising that to date no study has investigated IA in ADHD. IA has been studied and found altered in other neurodevelopmental disorders, such as autism spectrum disorder (ASD) and tic disorders [31,32]. In the latter study, some of the participants had comorbid ADHD, however whether this contributed to the findings was not tested.

**Fig 1:**
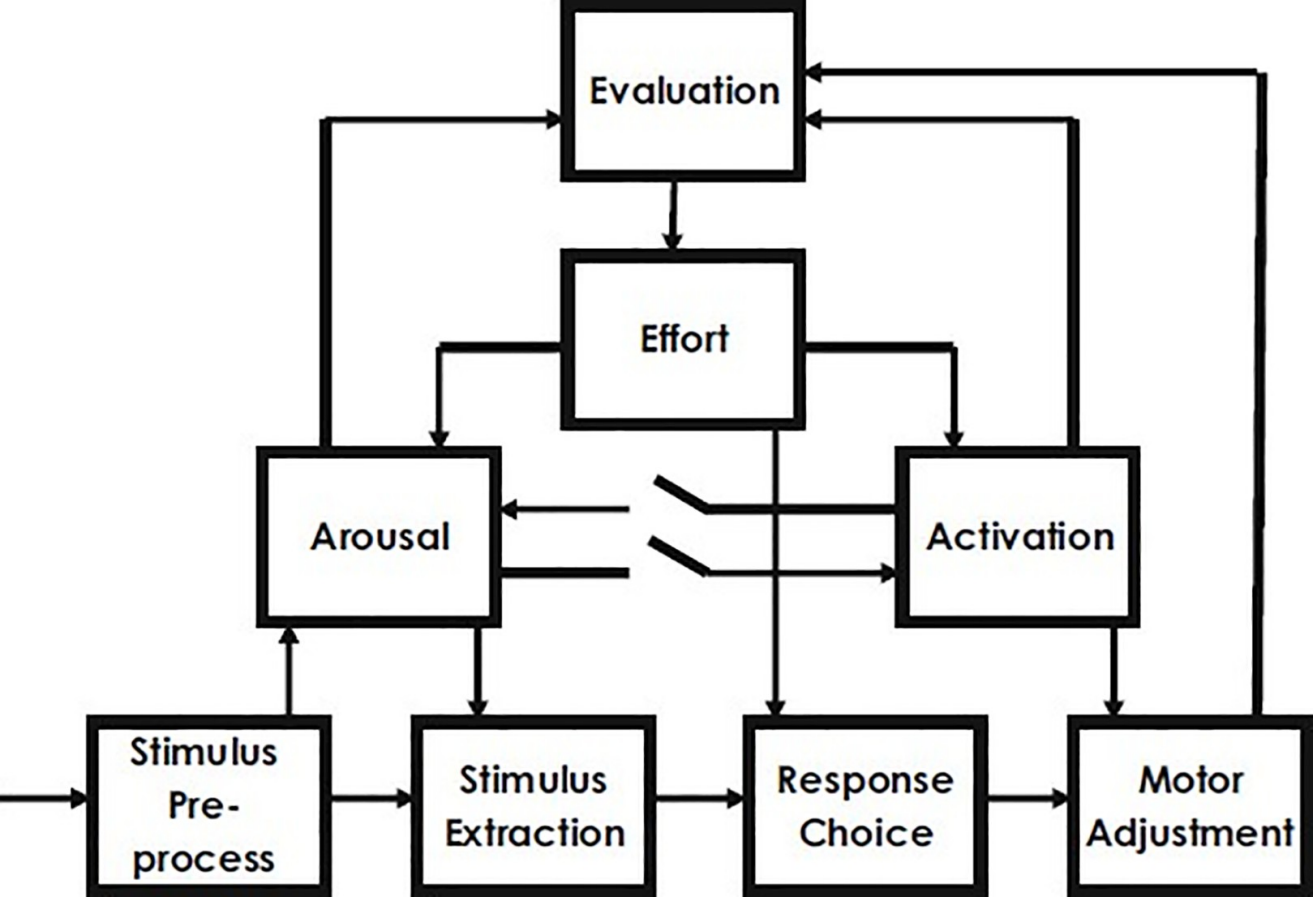
The cognitive energetic model of Sanders (1983).

The fact that IA has so far been neglected in ADHD research is even more surprising if one realizes that since the early 70’s dysfunctional autonomic regulation has been proposed to contribute to disturbed self-regulation in ADHD. Zentall defined the optimal stimulation theory and argued that the hyperactive behavior is an attempt to increase stimulation by the child with ADHD experiencing insufficient sensory stimulation [33]. Several researchers in the 80’s elaborated on this notion and proposed that ADHD is associated with hypo-arousal or deficient control of arousal levels [34]. In that same decade, Sergeant and van der Meere started to conduct a large number of studies applying the Cognitive Energetic Model: CEM [35] (see Fig 1.) of Sanders to ADHD [5,36], which led to the most recent and most elaborated model on arousal regulation deficits in ADHD, the state regulation deficit (SRD) account, which is nowadays one of the most influential ADHD models [5–7,36]. For a comprehensive explanation of the CEM and SRD, we would like to refer to existing reviews [5,6, 35,36]. In short, the main idea of the model is that behavior and task performance are dependent on the current energetic state of the organism (a distinction is made between phasic (called “arousal”) and tonic aspects of arousal (called “activation”), with arousal defined as a time-locked phasic physiological response to input, whereas activation refers to tonic readiness for action). Optimal performance requires optimal levels of arousal/activation. When the arousal or activation level is too low or too high, as scanned by an evaluation system, additional effort has to be implemented to counteract such a non-optimal energetic state (i.e. state regulation). The SRD account states that individuals with ADHD have difficulty doing so [5,6,36]. There is now ample behavioral and neurocognitive support from numerous studies for the SRD account. It is out of the scope of the current paper to provide an in-depth review of the evidence that supports the SRD account. For this, we would like to refer the reader to published studies, reviews and a meta-analysis [5–7, 36–40]. Together these studies have provided convincing evidence for a difficulty in children and adults with ADHD in adjusting arousal states. However, it is still not fully understood why this is the case.

According to the CEM, a prerequisite for effective state regulation is state monitoring: the evaluation system checks for discrepancies between the current and the required (target) arousal/activation state and in case of a discrepancy an individual can deliberately allocate extra effort in order to restore equilibrium. As can be seen in Fig 1., the CEM includes feedback loops from the energetic pools (arousal and activation) to the evaluation system. Hence, the CEM explicitly points to the importance of monitoring bodily signals for effective state regulation. If the ability to monitor bodily states and to become aware of these signals is impaired, logically, state regulation will be disrupted as well. We reasoned that if the basic ability of becoming aware of one’s bodily state, i.e. interoceptive awareness, is impaired, this may attribute to the weakened self-regulation skills and in particularly the difficulties in arousal regulation in ADHD. As this aspect has been completely overlooked in prior research, the aim of the current study was therefore to test for difficulties in this basic ability of interoceptive awareness in ADHD.

In the current study, we investigated IA in ADHD, by comparing adults with and without ADHD on objective and subjective indices of IA. IA was objectively measured by means of a well-validated heartbeat perception task [10,15,16], the Mental Tracking Method [41], in which participants are instructed to silently monitor their own cardiac activity during three separate intervals. This task is well-validated and the most frequently used task to assess interoceptive ability, which has been successfully applied in several research domains [10,16,18, 22]. Previous research has shown that this task is sensitive to interindividual differences in IA, and sensitive enough to uncover differences in IA in other neurodevelopmental disorders (e.g., autism spectrum disorder (ASD), tic disorder) [31,32]. Moreover, neuroimaging research further validated this task by showing enhanced activity of the anterior insula during the heartbeat perception task and local gray matter volume in the anterior insula being positively associated with IA [14]. In addition to the heartbeat task, we administered the Body Perception Questionnaire (BPQ) [42], which contains a subscale gauging the overall awareness of several bodily signals, which has frequently been applied in other experimental and clinical studies assessing interoceptive awareness (e.g., [43,44]). The reliability and validity of the awareness scale has been proven to be good as tested in three large independent samples collected in different countries [45].

Recapitulating, ADHD is considered a self-regulation disorder. IA has been shown to be of crucial importance for self-regulation. Weakened self-regulation in ADHD has for a long time been linked to arousal regulation difficulties. It is not known whether these may be due to impaired IA in ADHD. We therefore studied for the first time IA in adults with ADHD, by means of both objective and subjective measures. Based on existing findings, indicating impaired self-regulation and state regulation, we hypothesized lower IA in adults with ADHD.

## Method

### Participants

The study was approved by the local ethics committee of the Faculty of Psychological and Educational Sciences of Ghent University. Participants signed an informed consent. Twenty-five adults with ADHD between 19 and 37 years old (12 males) participated in this study. Data of one male participant were excluded because of the use of physical manipulations during the heartbeat perception task (see heartbeat perception task below). The results of the remaining 24 participants are reported (mean age: *M* = 23.46 (*SD* = 4.48), 12 males, one left-handed). The control group consisted of 23 typically developed adults, matched on age and sex (mean age: *M* = 23.57 (*SD* = 3.17), 13 males, four left-handed). Groups did not differ for age (*F*(1, 45) = 0.01, *p* = .926) or sex ratio (*χ^2^*(1) = 0.20, *p =* .654). The difference in IQ (controls: *M* = 111 (*SD* = 12); ADHD: *M* = 104 (*SD* = 13) between groups was marginally significant (*F*(1, 45) = 3.99, *p* = .052).

Individuals with ADHD were recruited through staff members, advertisements, self-support groups for ADHD, and a local database (adults with ADHD who participated in previous research). All adults with ADHD had a formal diagnosis established by a psychiatrist and completed a semi-structured clinical interview (DIVA, Diagnostisch Interview Voor ADHD bij Volwassenen 2.0) [46]. Adults with ADHD using stimulants (methylphenidate: MPH) were asked to stop this medication 48 hours prior to participation in the experiment. Seven participants with ADHD reported using MPH regularly, 12 only occasionally (e.g. for studying exams), 4 used it in the past but not anymore, and 1 person reported to never have used stimulants. Part of the controls were recruited via an online platform, called Experimetrix, at Ghent University, which is an online experiment scheduling system for students. Further for recruitment of controls and adults with ADHD, advertisements were placed on the website of ZitSTil, the Flemish knowledge and expertise centre for ADHD, and on online forums of popular magazines. Exclusion criteria for all participants were an estimated IQ below 80, history of brain-related illness or neurological disorder and a clinical diagnosis of depression or ASD. To asses IQ, both groups completed an abbreviated version [47] of the WAIS-IV (Wechsler Adult Intelligence Scale-IV) [48], except for the individuals with ADHD who were recruited through the local database since they had already completed the same abbreviated version of the WAIS-III in a previous study. Further, control participants had to score below clinical cut-offs in the attentive and hyperactive/impulsive domain, as evaluated by the Zelfrapportage Vragenlijst voor Aandachtsproblemen en Hyperactiviteit (ZVAH) [49], gauging presence of childhood or adulthood ADHD, in order to be included in the study.

In the ADHD group, 15 individuals exceeded the cutoff (> 46) of the Wechsler Utah Rating Scale (WURS) [50], a measure of presence of childhood ADHD, while no control participants exceeded the cutoff (controls: *M* = 16.7 (*SD* = 7.6); ADHD: *M* = 50.4 (*SD* = 13.5). In the ADHD group, according to the ZVAH, presence of childhood ADHD was confirmed for 20 participants (cutoff 6; 8 ADHD predominantly inattention, 12 ADHD combined subtype), while ADHD in adulthood was confirmed in 21 participants (cutoff 4; 7 ADHD predominantly inattention, 14 ADHD combined subtype). The scores on the Adult Self-Report (ASR) [51] revealed, not surprisingly, that the adult ADHD group scored significantly higher on the DSM oriented ADHD scale than the control group (*F*(1, 45) = 42.19, *p* < .001) (controls: *M* = 55.7 (*SD* = 6.7); ADHD: *M* = 74.3 (*SD* = 12.0)). Data on substance abuse, depression and anxiety were also collected via the ASR. No difference in substance abuse, as measured with the DSM oriented scale of the ASR, was observed between groups (*F*(1, 45) = 0.25, *p* = .620) (controls: *M* = 56.0 (*SD* = 6.9); ADHD: *M* = 56.9 (*SD* = 5.7)). The ADHD group scored significantly higher on the DSM oriented depression scale of the ASR compared to the control group (*F*(1, 45) = 12.55, *p* = .001) (controls: *M* = 52.7 (*SD* = 3.7); ADHD: *M* = 59.8 (*SD* = 9.0)), while the group difference on the anxiety scale did not reach significance (*F*(1, 45) = 2.89, *p* = 0.10) (controls: *M* = 53.1 (*SD* = 5.0); ADHD: *M* = 55.8 (*SD* = 6.0)).

Several factors that have previously been shown to affect IA and could confound the results were assessed. First, both anxiety and depression have been shown to be (differently) related to IA [52–54]. As mentioned, adults with ADHD scored higher on depression symptoms, but no difference in anxiety symptoms was noticed. Second, alexithymia, which reflects difficulty in identifying and describing feelings and is characterized by externally oriented thinking, has been shown to be negatively correlated with IA [55]. Alexithymia was measured with the Toronto Alexithymia Scale (TAS-20) [56]. The ADHD group scored significantly higher on the TAS-20 in comparison to the control group (*F*(1, 45) = 7.93, *p* = .007) (controls: *M* = 40.1 (*SD* = 7.0); ADHD: *M* = 48.6 (*SD* = 12.7)).

### Measurements of IA

#### Heartbeat perception task

The Mental Tracking Method [41], a well-validated task with good psychometric values [57] and repeatedly used to measure IA [10,16,18, 22], was chosen as an objective measure of IA. The heartbeat perception task was programmed using E-Prime 2.0 software (http://www.pstnet.com/products/e-prime/) and presented on a 19-inch CRT monitor with 640x480 screen resolution and a 60 Hz refresh rate. All sessions took place during daytime for all participants.

In accord with earlier studies using the Mental Tracking method [16,22,41], participants were instructed to focus on their own cardiac activity and silently count the number of heartbeats during three separate intervals. There were three different intervals (25s, 35s, 45s) and the order of presentation of these three intervals was random. Participants were explained beforehand that there would be three intervals during which they had to silently count their own heartbeats. The length of the intervals was not communicated to the participants. They were explained that an interval was indicated by a start and stop tone (coming from the PC speaker) corresponding with the beginning and end of an interval, and that they had to verbally report the number of counted heartbeats. Every trial started with a question on the screen, asking whether participants were ready. After this prompt, the start sound was presented together with a blank screen. After the stop signal, participants verbally reported the number of counted heartbeats during a resting period of 30 s, after which the prompt of the following interval was presented anew. The reported number was written down by the experimenter out of view of the participant. Participants did not receive feedback on their performance. Importantly, participants were instructed not to use physical manipulations (e.g. taking their pulse) to ease the counting and the experimenter monitored the participants through a camera.

A heartbeat perception score was derived in keeping with previous studies [16,22,41]: per interval a difference score of the number of recorded and counted heartbeats was calculated. These difference scores were then divided by the number of recorded heartbeats, subtracted from 1, summed and averaged by the number of intervals. Due to this formula: 1/3 Σ (1 –(|recorded heartbeats–counted heartbeats|) / recorded heartbeats), the heartbeat perception score could vary between 0 and 1, with higher scores indicating higher IA and thus a small difference between counted and recorded heartbeats.

The heartbeat perception task was administered twice, at the beginning and end of the total testing session (see procedure for details). A mean heartbeat perception score was calculated by summing both heartbeat perception scores (acquired at the beginning and end of the testing session), and dividing that sum by two (hence based on in total 6 heartbeat counting intervals).

The electrocardiogram was recorded via two external electrodes from the Biosemi ActiveTwo system (Biosemi, Amsterdam, The Netherlands); one electrode was placed on the left lower rib cage and one electrode was placed on the left upper rib cage. R-waves were counted offline by means of a custom-made R-top algorithm in Brain Vision Analyzer 2 software.

#### Body Perception Questionnaire (BPQ)

Participants completed the Dutch translation of the BPQ [42], a subjective self-report measure of IA. We used an official back-translated version, approved by the authors of the original BPQ (translated version by Godefroid, Dhar & Wiersema, available at stevenporges.com). This questionnaire consists of four different subscales with a total of 96 items. The subscale of interest for our study was the awareness subscale, which consists of 45 items (Cronbach’s α: .97 for both groups), questioning how aware participants are of their autonomic signals (e.g., swallowing frequently, how hard my heart is beating). Research has shown good reliability and validity measures for the awareness scale [45]. Although this subscale was of main interest for our study, the other subscales (stress response; reactivity of the autonomic nervous system; stress style) of the BPQ were included to check for specificity. Items are rated on a five-point Likert scale, ranging from 1 (never) to 5 (always). The mean score of each subscale was obtained by summing all responses and dividing the sum by the number of items in the subscale.

### Procedure

Participants were seated in a sound-attenuated and dimly lit room, sitting approximately 60 cm in front of the computer screen. Each participant signed an informed consent prior to participation in the experiment and received monetary compensation for their participation. Then participants were explained about the tasks that would follow. This study was part of a larger experimental set-up. Two other behavioral tasks with a total duration of 50 min were administered in between both administrations of the heartbeat perception task; the results of these tasks will be reported elsewhere. Verbal as well as written instructions were given prior to the start of each task. This study was approved by the local ethics committee.

### Data analysis

ANOVAs with group (ADHD vs. control) as between-subjects factor were performed to compare performance between groups on the heartbeat perception task, and the subjective measure of IA as indexed by the mean score of the awareness subscale of the BPQ. To check for specificity, we also performed ANOVAs on the mean scores of the other subscales of the BPQ. Finally, links between the IA indices and symptoms of anxiety, depression, and alexithymia were explored by additional correlational analyses.

## Results

### Performance on the heartbeat perception task

For both groups, the heartbeat perception score obtained at the beginning of the session correlated significantly with the heartbeat perception score acquired at the end (ADHD: *r* = .80, *p* < .001; control: *r* = .92, *p* < .001), indicating that the estimate of IA was reliable. Moreover, mean heartbeat perception scores obtained were comparable to previous studies. For the ADHD group, the mean heartbeat perception score was .81 (range: .45 - .97), while it was .83 (range: .45 - .97) in the control group. The between-group comparison in mean heartbeat perception score yielded no significant results (*F*(1, 45) = 0.23, *p* = .634), with a striking similar standard deviation (.15 for both groups). The Data are reported in Table 1, separately for the two groups.

**Table 1:** Scores on the objective and subjective measure of interoceptive awareness, separated per group.

	ADHD	Control
Objective measure		
Heartbeat perception score 1	.80 (.16)	.81 (.17)
Heartbeat perception score 2	.83 (.15)	.86 (.14)
Mean heartbeat perception score	.81 (.15)	.83 (.15)
Average heart rate (bpm)	68.39 (9.59)	68.81 (10.76)
Subjective measure		
Awareness	2.35 (0.80)	2.08 (0.73)
Stress Response	2.85 (0.79)	2.20 (0.55) *
Reactivity of the ANS	1.68 (0.43)	1.40 (0.30) *
Stress Style	2.62 (0.57)	2.29 (0.35) *

*Note*. Values are shown as means (*SD*). bpm = beats per minute.

* indicates a significant difference.

As previous research has indicated worse IA as indexed by the heartbeat perception task in females versus males [57], an additional ANOVA was performed with group (ADHD vs. control) and gender as between-subjects factors. This did not change the findings, as neither the main group effect (*F*(1, 43) = 0.22, *p* = .641), nor the group by gender effect (*F*(1, 43) = 0.02, *p* = .902) was significant. The main effect of gender was also not significant (*F*(1, 43) = 0.03, *p* = .859).

No significant correlations between alexithymia, anxiety or depression scores and the mean heartbeat perception score were observed, neither in the ADHD group (alexithymia: *r* = .05, *p* = .805; depression: *r* = -.04, *p* = .866; anxiety: *r* = -.10, *p* = .649), nor in the control group (alexithymia: *r* = -.18, *p* = .412; depression: *r* = .02, *p* = .922; anxiety: *r* = -.05, *p* = .815).

### Body Perception Questionnaire (BPQ)

The between-group comparison of the score obtained on the awareness subscale yielded no significant results (*F*(1, 45) = 1.41, *p* = .24). Although groups did not differ on the subscale measuring awareness, adults with ADHD scored higher on the other three subscales of the BPQ, namely stress response (*F*(1, 45) = 10.84, *p* = .002), reactivity of the autonomic nervous system (*F*(1, 45) = 6.81, *p* = .012), and stress style (*F*(1, 45) = 5.85, *p* = .020, see Table 1).

No significant correlations between alexithymia, anxiety or depression scores and the score on the awareness subscale were found, neither in the ADHD group (alexithymia: *r* = .14, *p* = .518; depression: *r* = .16, *p* = .467; anxiety: *r* = .20, *p* = .356), nor in the control group (alexithymia: *r* = -.13, *p* = .546; depression: *r* = .11, *p* = .604; anxiety: *r* = .27, *p* = .206).

## Discussion

As information regarding the momentary bodily state is of crucial importance for effective self-regulation, and in particular state regulation, ADHD may be associated with lower IA. Surprisingly, this hypothesis has not yet been tested. The aim of the present study was thus to investigate IA in adult ADHD by means of an objective and subjective measure. The heartbeat perception task was administered to gain an objective measure of IA, while a questionnaire was used (the awareness subscale of the BPQ) to assess a self-report measure of IA. Performance on the heartbeat perception task was strikingly similar in adults with ADHD compared to healthy controls. Moreover, adults with ADHD and typically developed adults also did not differ on the self-report measure of IA. Findings therefore suggest preserved monitoring of bodily state in adult ADHD, which tentatively suggests that the state regulation deficit and related self-regulatory difficulties in ADHD may not be due to an inability to monitor the current bodily state.

Several possible reasons for this null-result can be formulated. For instance, it could be related to the paradigm used in the current study. However, this suggestion is doubtful since the heartbeat perception task that we applied is a well validated and widely used paradigm to assess IA in different domains [10,15,16,18, 22] and has previously been shown to be sensitive enough to uncover differences in IA in other developmental disorders (autism spectrum disorder, tic disorder) [31,32] and other clinical groups (anxiety and depression) [52]. Moreover, it has been validated in neuroimaging research that showed enhanced activity of the anterior insula during this task [14]. Also, the scores are comparable to scores from previous studies using the same paradigm [16,22] and these scores furthermore indicate that both groups were able to perform well above chance level. Furthermore, corroborating our finding is the striking similarity in variance of the mean heartbeat perception scores between both groups. In addition, comparing only the heartbeat perception score acquired at the beginning of the session between groups, gave the same null result, excluding the possibility that learning effects across both sessions of the task may explain the findings. Also, important to note is that all necessary steps were taken to rule out using any tricks to estimate one’s heartbeats (see methods section).

Moreover, and importantly, preserved IA in ADHD was confirmed by the self-report measure of IA, the awareness scale of the BPQ [42]. The BPQ assesses sensitivity of interoceptive signals in daily life. The findings indicate also no difference between groups for this index. Recent research has shown good reliability and validity indices for the awareness scale, as tested in three large independent samples collected in different countries [45]. In order to stay as close as possible to the original BPQ, we used an official back-translated Dutch version, approved by the authors of the original BPQ. Nevertheless, future research may be needed to validate the Dutch version of the BPQ as used in the current study.

Other factors previously shown to be related to IA could have potentially obscured our findings. Alexithymia, anxiety and depression have been (differently) related to IA [52–55]. In the current study, groups did not significantly differ for symptoms of anxiety, but adults with ADHD reported more symptoms of depression and alexithymia. However, correlations between indices of these factors and scores on both measures of IA were negligible in both groups, suggesting no association between IA and those constructs in our sample. Moreover, as alexithymia has previously been shown to be negatively associated with IA [55], higher alexithymia symptoms in ADHD would result in lower IA in adults with ADHD and cannot explain the absence of a difference in IA between groups. The same reasoning holds for the elevated depression symptoms in ADHD. For mild to moderate levels of symptoms of depression, a negative relation has been reported between IA and symptoms of depression [58]. Hence, more symptoms of depression within our ADHD sample could contribute to lower IA in ADHD, but cannot explain similar IA ability. Anxiety on the other hand has been positively correlated with IA [53]. Thus, elevated levels of anxiety could have resulted in elevated IA in the ADHD group. This factor is however highly unlikely to explain the findings, because the groups did not differ on anxiety symptoms and anxiety was not found to be correlated with IA.

ADHD is a heterogeneous neurodevelopmental disorder and we are aware that the findings may not generalize to all individuals with ADHD. Both adults with ADHD predominantly inattentive and combined subtypes were included in the study. It would be of interest to test for differences in IA between subgroups or subtypes of ADHD, however, in our study, this was not possible as separate groups were not large enough. Also, both men and women with ADHD were included. Groups were however carefully matched on gender and additional analyses showed no influence of gender. With regard to severity of ADHD symptoms, we feel confident that we tested a representative sample of adults with ADHD. All participants had a formal clinical diagnosis provided by a multidisciplinary team including a psychiatrist and this diagnosis was confirmed by a clinical interview. Also, although adults with ADHD did not differ on the awareness subscale of the BPQ, they were found to have more difficulties in autonomic reactivity, stress response and stress style (the other subscales of the BPQ), which is in line with previous research in adults with ADHD, in which elevated physiological stress responses and higher self-reported stress were observed [59–61]. Nonetheless, as to our knowledge this is the first study on IA in ADHD, further research is warranted to replicate our findings in other samples as well as in children with ADHD, preferably with alternative measures as well, before final conclusions on IA in ADHD can be formulated.

The findings suggest that the basic skill of IA is intact in ADHD during a simple heartbeat perception task, but this does not rule out the possibility that becoming aware of bodily signals might be disrupted in other situations in daily life, perhaps as a result of reduced attention or distraction. In other words, in the current situation, when distraction was very limited, adults with ADHD were equally well in monitoring their interoceptive signals, but it has to be tested whether this is still the case when other (distracting) events are happening and additional tasks have to performed, which often is the case in daily life. Similarly, our finding of intact basic ability to become aware of bodily signals, does not exclude the possibility that during situations that require state regulation, this ability is not disrupted. This could be addressed in future research. Alternatively, it could be that IA in ADHD is preserved also in other situations, but that interoceptive information is wrongly applied or interpreted. In other words, individuals with ADHD might be able to monitor the momentary body state but are not able to use the available information sufficiently. This can be further explored in future studies. The finding of preserved IA in ADHD as measured in the current study, instigates the debate on whether the self-regulatory difficulties in ADHD reflect a difficulty in allocating the required effort or are related to a general altered motivational style [6,62]. Further research is needed to examine these hypotheses.

Some limitations have to be mentioned. First, as this is the first study investigating IA in ADHD and ADHD is known to be a heterogeneous disorder, generalization to other samples with ADHD is difficult. Replication of these findings is thus warranted. Second, although the heartbeat perception task we used is a well-validated task that has been extensively applied in previous research, it would be informative if results hold for other heartbeat perception paradigms, such as a heartbeat discrimination task. Third, individuals with ADHD with and without use of medication (methylphenidate) were included in the study. Although we asked participants to stop their medication 48 hours prior to participation, in line with a lot of existing studies, it cannot be fully excluded that medication use may have influenced our findings. An exploratory data check did however not indicate a difference in IA scores between individuals with ADHD who were or were not taking methylphenidate in daily life.

In summary, following recent research indicating IA to contribute to self-regulation and previous research inspired by the SRD model that unequivocally provided support for deficient regulation of energetic state in ADHD, the hypothesis was put forward that the ability to monitor the momentary bodily state may be impaired in ADHD, a hypothesis that had been overlooked in previous research. However, no support was rendered for altered IA in adults with ADHD. The finding of preserved monitoring of bodily state tentatively suggests that self-regulatory difficulties in ADHD may not be related to a lack of awareness about the current bodily state.
